# Dose-Distribution-Driven PET Image-Based Outcome Prediction (DDD-PIOP): A Deep Learning Study for Oropharyngeal Cancer IMRT Application

**DOI:** 10.3389/fonc.2020.01592

**Published:** 2020-08-18

**Authors:** Chunhao Wang, Chenyang Liu, Yushi Chang, Kyle Lafata, Yunfeng Cui, Jiahan Zhang, Yang Sheng, Yvonne Mowery, David Brizel, Fang-Fang Yin

**Affiliations:** ^1^Department of Radiation Oncology, Duke University Medical Center, Durham, CA, United States; ^2^Medical Physics Graduate Program, Duke Kunshan University, Kunshan, China

**Keywords:** Positron Emission Tomography, deep learning, oropharangeal cancer, IMRT (intensity modulated radiation therapy), outcome prediction

## Abstract

**Purpose:**

To develop a deep learning-based AI agent, DDD-PIOP (Dose-Distribution-Driven PET Image Outcome Prediction), for predicting ^18^FDG-PET image outcomes of oropharyngeal cancer (OPC) in response to intensity-modulated radiation therapy (IMRT).

**Methods:**

DDD-PIOP uses pre-radiotherapy ^18^FDG-PET/CT images and the planned spatial dose distribution as the inputs, and it predicts the ^18^FDG-PET image outcomes in response to the planned IMRT delivery. This AI agent centralizes a customized convolutional neural network (CNN) as a deep learning approach, and it incorporates a few designs to enhance prediction accuracy. 66 OPC patients who received IMRT treatment on a sequential boost regime (2 Gy/daily fraction) were studied for DDD-PIOP development. 61 patients were used for AI agent training/validation, and the remaining five were used as independent tests. To evaluate the developed AI agent’s performance, the predicted mean standardized uptake values (SUVs) of gross tumor volume (GTV) and clinical target volume (CTV) were compared with the ground truth values. Overall SUV distribution accuracy was evaluated by gamma test passing rates under different criteria.

**Results:**

The developed DDD-PIOP successfully generated ^18^FDG-PET image outcome predictions for five test patients. The predicted mean SUV values of GTV/CTV were 3.50/1.41, which were close to the ground-truth values of 3.57/1.51. In 2D-based gamma tests, the average passing rate was 92.1% using 5%/10 mm criteria, which was improved to 95.9%/93.2% when focusing on GTV/CTV regions. 3D gamma test passing rates were 98.7% using 5%/10 mm criteria, and the corresponding GTV/CTV results were 99.8%/99.4%.

**Conclusion:**

The reported results suggest that the developed AI agent DDD-PIOP successfully predicted ^18^FDG-PET image outcomes with high quantitative accuracy. The generated voxel-based image outcome predictions could be used for treatment planning optimization prior to radiation delivery for the best individual-based outcome.

## Introduction

The oropharynx is one of the most common sites of origin of head-and-neck cancer. Nearly 20,000 new cases of oropharyngeal cancer (OPC) are diagnosed annually in the United States (1, 2). Radiotherapy (RT) with or without concurrent chemotherapy constitutes one of the standard treatment options for OPC. Compared to conventional 3D radiation treatment, intensity-modulated radiation therapy (IMRT) has demonstrated an improved therapeutic index with similar rates of tumor control and reduced toxicity to the organs-at-risk (OARs) (3). Consequently, IMRT has become the standard approach for delivering RT in OPC.

Positron Emission Tomography (PET) provides information on the metabolic status of a tumor and plays a crucial role in staging and response assessment to RT for OPC (4–10). PET has been also been used for primary and nodule target volume delineation for treatment planning. An ongoing area of investigation is the use of PET for assessing interim treatment effect for adaptive radiotherapy implementation (11–13). Although PET scans contain valuable information for adaptive radiotherapy in a longitudinal treatment course, they introduce extra ionizing radiation risk (>10 mSv) and add significant extra cost and effort (14, 15). Furthermore, the optimal time point of an interim PET is unknown. As a result, image-based outcome prediction prior to the radiotherapy delivery is conceptually appealing: based on the prediction, one can optimize many radiation plan parameters to maximize the therapeutic outcomes and address potential issues that indicated by the prediction.

PET/CT images contain substantial predictive information in terms of functional metabolic rate change (16, 17). Recently, radiomics analysis that extracts high throughput features from medical images have been reported for predictions of progression (18), distant metastasis development (19), and treatment outcome (20). With the rapid growth of computation power, deep learning techniques have become a practical approach to the implementation of artificial intelligence (AI). As a representative deep learning technique, CNN is one such technique for making treatment outcome predictions (21, 22). However, image-based radiotherapy outcome predictions remain challenging, and the incorporation of plan-specific information has yet to be investigated.

In this work, we aim to develop a deep learning-based AI agent for predicting PET image changes that occur in response to specific oropharyngeal IMRT treatment plans. The developed AI agent, Dose-Distribution-Driven PET Image-Based Outcome Prediction (DDD-PIOP), utilizes pre-treatment PET/CT images and the planned dose distribution as the patient-specific source information and predicts a PET image as the outcome of the planned dose delivery. DDD-PIOP could potentially be further developed as a powerful tool for physiologically-based IMRT dose alteration (“dose painting”): given a radiation plan dose distribution with the associated PET image outcome prediction, the planner(s) can adjust the target volume definitions, target dose prescription levels, and OARs dose constraints on a voxel level to obtain an updated plan with possible iterative process to maximize individual IMRT outcome effects.

## Materials and Methods

### DDD-PIOP Design

**FIGURE 1:**
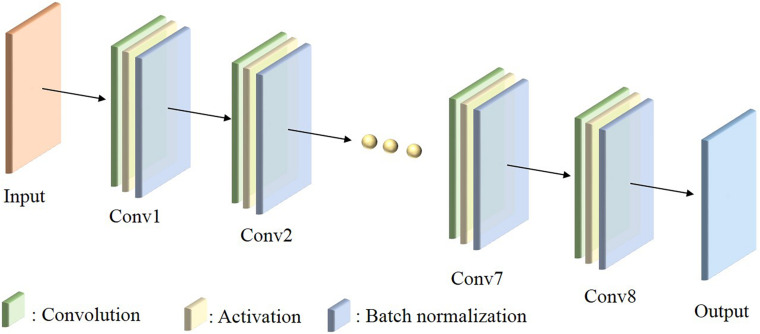
The illustration of CNN architecture in DDD-PIOP.

The developed DDD-PIOP AI agent centralizes a custom-build CNN as demonstrated in Figure 1. As illustrated, the constructed CNN has a total of 8 convolutional layers. Multiple filters are applied in each convolutional layer for screening predictive features. All filters are designed with a kernel size of 3 × 3. As the network goes deeper, the number of filters in each convolution layer increases from 32 to 256, and then collapses to 1. The image dimension remains unchanged from the original ones of input. Such a design enables the exploration of hidden hierarchical features and the associations of spatial information with explored features. Additionally, each convolution layer is followed by an activation function. Rectifier linear unit (ReLU) was chosen as the activation function due to its high computational efficiency and smaller likelihood of gradient vanishing.

### DDD-PIOP Training

**FIGURE 2:**
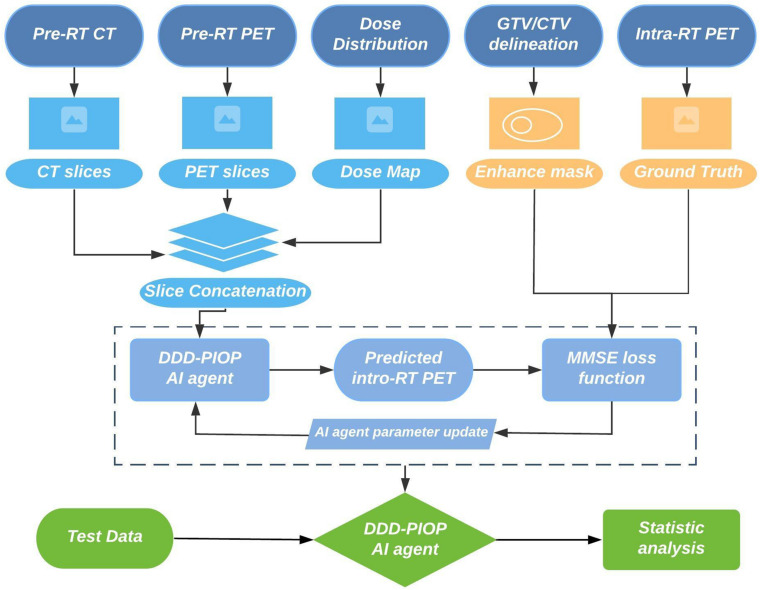
DDD-PIOP training workflow.

DDD-PIOP training process involved multi-scale patient resources. Figure 2 illustrates a brief workflow of DDD-PIOP training. Specifically, pre-radiotherapy (pre-RT) PET/CT images and planned dose distributions are used as the CNN inputs. The planned dose distributions were derived from the radiotherapy dose DICOM. 2D axial slices of pre-RT PET/CT images and the resampled dose map are concatenated as a 3D matrix for single channel input. After CNN implementation, a 2D axial PET image that predicts treatment response can be generated as the output. During DDD-PIOP training, the loss function is designed as a modified mean square error (MMSE) between the CNN output and the ground truth images from the training cases. Since gross tumor volume (GTV) and clinical target volume (CTV) regions are of more attentions as their primary roles in IMRT, these two regions were prioritized in MMSE in Equation (1):

(1) $$L_{l\,o\,s\,s}=\sum_{i,j=0}^{i,j}1_{i,j}^{n\,o\,n}\,{\left(p_{i,j}-g_{i,j}\right)}^{2}+w_{C\,T\,V}\cdot \sum_{i,j=0}^{i,j}1_{i,j}^{C\,T\,V}\,{\left(p_{i,j}-g_{i,j}\right)}^{2}+w_{G\,T\,V}\cdot \sum_{i,j=0}^{i,j}1_{i,j}^{G\,T\,V}\,{\left(p_{i,j}-g_{i,j}\right)}^{2}$$

where *p*_*i,j*_ is the voxel SUV in the predicted PET image and *g*_*i,j*_ is the voxel SUV in the ground truth PET images. *w*_*GTV*_ /*w*_*CTV*_ are factors for prioritized GTV/CTV penalties and were set as 6.0/3.0 empirically. $1_{i,j}^{G\,T\,V}$, $1_{i,j}^{C\,T\,V}$, and $1_{i,j}^{n\,o\,n}$ are binary regions-of-interest (ROI) masks that were derived from the IMRT structure DICOM file. To account for scale dispersion, the input images (PET/CT and dose distribution) were normalized to [0,1] range. Prior to the final output, the prediction PET images were restored to the original range using the adopted input normalization values.

DDD-PIOP was implemented in Tensorflow and Keras environment using a NVIDIA (NVIDIA Inc., Santa Clara, CA, United States) GTX1060Ti graphic card. The Adam optimization algorithm was utilized for adaptive learning rate. The training process had 50 epochs and the overall training time was about 1 h.

### Patients

DDD-PIOP was developed from scans that were prospectively obtained from patients who were enrolled on an IRB approved trial of PET imaging. A total of 66 eligible OPC patients who received 70 Gy IMRT delivery with a sequential boost regime (2 Gy/daily fraction) were studied. Each patient received a pre-RT PET/CT scan and a 2nd PET/CT scan after 20 Gy delivery (intra-RT scan) for the adaptive purpose. The intra-RT PET image volumes were used for DDD-PIOP training while pre-RT PET/CT image volumes and 20 Gy dose distribution were used as the inputs.

All PET/CT images were acquired by a clinical PET/CT scanner (Siemens, Erlangen, Germany). PET images were acquired by 400 × 400 matrix size in a standard field of view (FOV) of 54 cm, and slice thickness was 2 mm. CT images were acquired by 512 × 512 matrix size in an extended FOV of 65 cm, and slice thickness was 3 mm. A small FOV (30 cm) CT volume was reconstructed from the standard acquisition for contouring purpose. All PET images were reconstructed by the ordered subset expectation maximization (OSEM) algorithm with attenuation corrections using the CT volumetric information. IMRT planning was performed on the extended FOV CT volume using Eclipse system (Varian Medical System, Palo Alto, CA, United States). Dose calculation was implemented in 2.5 mm grid size. Both PET images and dose distributions were resampled to the CT volume grid size for DDD-PIOP execution.

The cohort was randomly divided into two subgroups: 61 patients were used for DDD-PIOP training/validation group and the remaining five patients were used as independent tests. Prior to the DDD-PIOP training, the intra-RT PET/CT image volumes were registered to the pre-RT PET/CT image volumes using deformable multi-pass registration algorithm and Velocity software (Varian Medical System, Palo Alto, CA, United States).

### DDD-PIOP Evaluation

The accuracy of image outcome prediction was evaluated using the 5 independent test patients. The predicted intra-RT PET images were first compared with the ground truth intra-RT PET images by qualitative visual checks. Quantitative accuracy was evaluated by regional mean SUV value comparisons. Specifically, mean SUV values inside GTV/CTV were compared with the corresponding ground truth values. In addition, mean SUV value in high ^18^FDG uptake region were also evaluated. Such high uptake region was determined by the Otsu’s method as a histogram-based segmentation method within GTV (23, 24).

In addition to regional mean SUV comparison, SUV distribution accuracy was evaluated by SUV value gamma test passing rates with different criteria. As defined in equation (2), gamma test incorporates both SUV value differences and SUV distribution shifts into considerations (25).

(2) $$\gamma =m\,i\,n\,\left(\sqrt{{\left(\frac{\Delta \,S\,U\,V}{\Delta \,S\,U\,V_{t}}\right)}^{2}+{\left(\frac{\Delta \,d}{\Delta \,d_{t}}\right)}^{2}}\right)$$

where Δ*SUV* is SUV difference and Δ*d* is the SUV distribution shift. Δ*SUV*_*t*_ is the acceptance criteria for SUV uptake and Δ*d*_*t*_ is the acceptance criteria for distance to agreement (DTA). The passing criterion of γ value at each pixel is set to 1, and the percentage of voxels that pass gamma test were reported as indicators of prediction accuracy. Different gamma passing criteria were evaluated to study the differences of GTV/CTV and whole body results. Both 2D and 3D gamma tests were reported.

## Results

**FIGURE 3:**
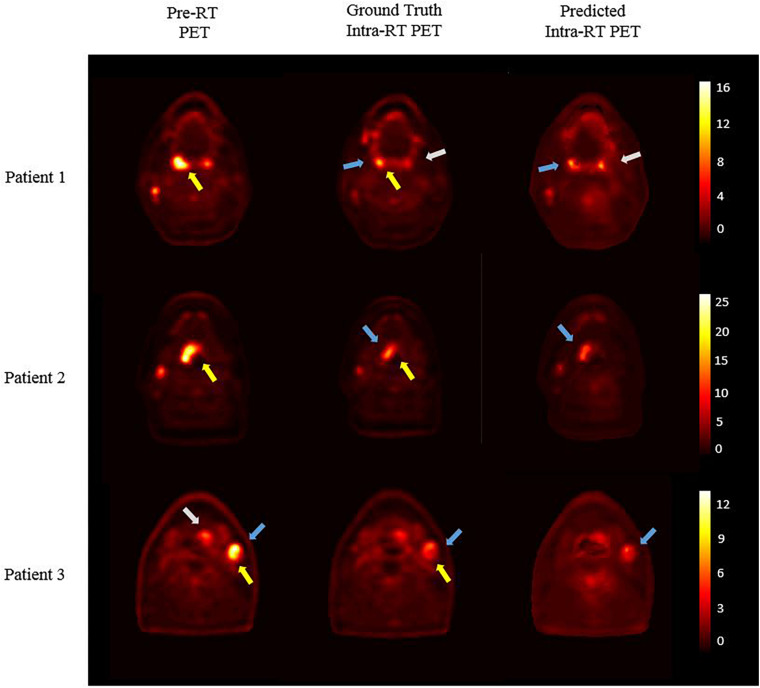
DDD-DIOP results of three test patients. Left column: pre-RT PET; middle column: ground truth intra-RT PET; right column: predicted intra-RT PET. Yellow arrows: SUV reduction after 20 Gy as treatment response; Blue and white arrows: DDD-PIOP predictions captured ground-truth results.

Figure 3 presents three independent test patients’ results. The left column shows pre-RT PET images, and corresponding ground truth intra-RT PET images are shown in the middle column. As indicated by yellow arrows, treatment response can be observed with reduced SUV hotspot sizes and decreased SUV intensities after IMRT delivery. The right column shows the predicted intra-RT PET images from DDD-PIOP. The predicted images captured overall PET image appearance successfully without noticeable artificial manipulation marks. The hotspots within the radiation treatment region (indicated by blue arrows) of the predicted intra-RT PET images demonstrate similar spatial patterns and comparable SUV intensity ranges in reference to the ground truth intra-RT PET images. Subtle variations in SUV hotspots are predicted by the DDD-DIOP. As an example, in patient 1, a residual hotspot (indicated by the white arrows) after radiotherapy is accurately predicted in terms of spatial location. SUV distributions other than hotspot regions were also successfully predicted with observable and yet minor discrepancies.

Table 1 shows the quantitative comparison of mean SUV values. In GTV and CTV regions, the values from the predicted image volumes were close to the corresponding ground truth values, and the absolute SUV differences were 0.10 or less. These small differences would not lead to clinically significant differences in metabolic volume definition based on SUV thresholding. In the identified high ^18^FDG uptake regions, the predicted value was also close to the ground truth value, and the prediction tended to underestimate the SUV results. No statistical tests were included due to limited test case number (=5).

**TABLE 1:** The mean SUV value prediction results inside GTV region, CTV region, and high-uptake region in comparison with the ground truth.

	GTV-region	CTV-region	High-uptake region
Predicted mean SUV value	3.50	1.41	4.63
Ground truth mean SUV value	3.57	1.51	4.92

**FIGURE 4:**
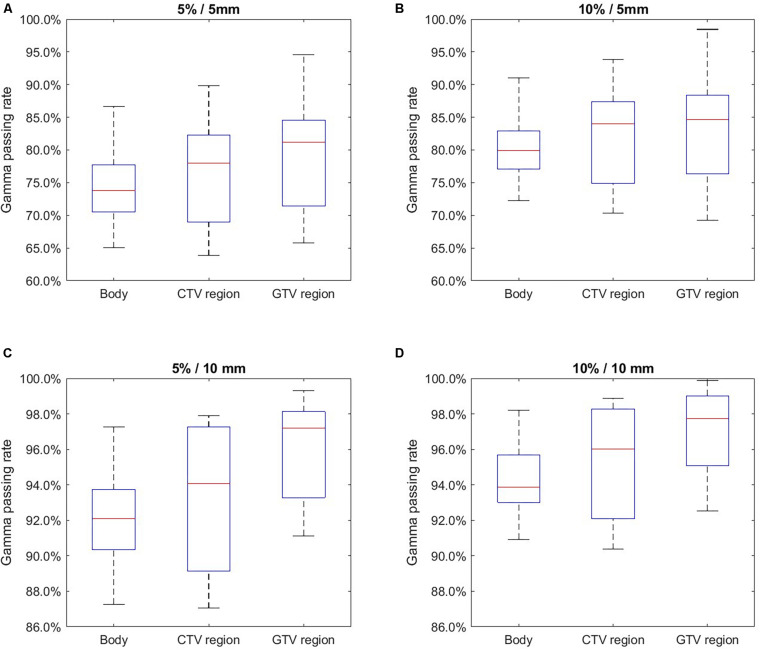
2D gamma test passing rate evaluation results using different acceptance criteria with 20–80% whiskers. **(A)** 5%/5 mm; **(B)** 10%/5 mm; **(C)** 5%/10 mm; **(D)** 10%/10 mm.

Figure 4 and Table 2 summarize the 2D gamma test results with different gamma test criteria (ΔSUV/DTA). As shown, the passing rates were improved with more generous test criteria. When whole body area was evaluated, the average gamma test passing rate was 74.5% using the strictest criterion (5%/5 mm). This result is acceptable and yet less optimal. With the 5%/5 mm criterion, the passing rates were improved in GTV and CTV regions with higher SUV intensities. This trend also occurs in the tests with other criteria and thus serves during DDD-PIOP training. The best result was obtained with the 10%/10 mm criterion, where the passing rate in the GTV region was 97.0%. As gamma test criteria became looser from 5%/5 mm to 10%/10 mm, the difference between CTV/GTV and BODY results were stable, and BODY results demonstrated more obvious improvements.

**TABLE 2:** The average 2D gamma test passing rate results using different acceptance criteria and evaluation ranges.

Gamma test criteria	Whole body (%)	CTV region (%)	GTV region (%)
5%/5 mm	74.5	76.4	79.2
10%/5 mm	80.4	82.0	83.2
5%/10 mm	92.1	93.2	95.9
10%/10 mm	94.3	95.2	97.0

**FIGURE 5:**
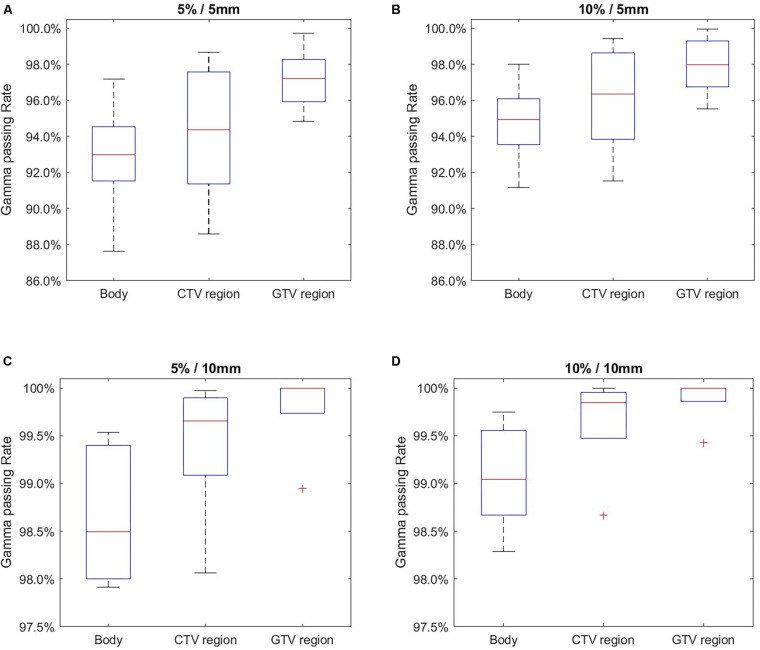
3D gamma test passing rate evaluation results using different acceptance criteria with 20–80% whiskers. **(A)** 5 %/5 mm; **(B)** 10%/5 mm; **(C)** 5%/10 mm; **(D)** 10%/10 mm.

Figure 5 and Table 3 summarize the 3D gamma test passing rate results. In general, 3D gamma test results were better than 2D gamma test results. At the strictest criterion (5%/5 mm), the 3D gamma test result reported a passing rate of 92.9% in whole body evaluation. A similar trend was also presented in 3D gamma test evaluation: higher gamma test passing rates in the lesion region. When using the 10%/10 mm criterion, the DDD-PIOP achieved the best 3D gamma pass rate of 99.9% in the GTV region. Like the 2D gamma test results, BODY results showed more obvious improvements when using looser gamma test criteria.

**TABLE 3:** The average 3D gamma test passing rate results using different acceptance criteria and evaluation ranges.

Gamma test criteria	Whole body (%)	CTV region (%)	GTV region (%)
5%/5 mm	92.9	94.2	97.2
10%/5 mm	94.8	96.1	97.9
5%/10 mm	98.7	99.4	99.8
10%/10 mm	99.1	99.6	99.9

## Discussion

In this work, we developed an AI agent DDD-PIOP for OPC IMRT treatment outcome prediction. The goal of the DDD-PIOP is to predict an interim treatment PET image using pre-treatment PET/CT images and the spatial dose distribution. Image-based outcome predictions by DDD-PIOP are promising with acceptable quantitative results. Specifically, DDD-PIOP achieved high accuracy in predicting GTV/CTV volumes and their sub-volumes with high SUV values. The improved results in GTV/CTV could be attributed to the contour information as plan-specific annotations in DDD-PIOP inputs. Since high SUV regions are commonly used for treatment target definition, the current DDD-PIOP design enables its suitability for adaptive planning (and possible IMRT dose painting with future development) based on target definition. In future works, it will be of great interest to investigate normal tissue toxicity reduction in DDD-PIOP.

A good training design and validation implementation is crucial for optimal CNN performance. In the current version of DDD-PIOP, the loss function for CNN training is MMSE that emphasizes GTV/CTV region results. MMSE did not encounter the common non-convergence problem during the CNN training. The evaluation of DDD-PIOP needs to consider many factors, including PET’s intrinsic uncertainty, PET image acquisition resolution, PET-CT QA protocol, deformable registration uncertainty (26), and the clinical use of SUV in metabolic volume definition. The adopted gamma test criteria were designed with the considerations of the aforementioned aspects. More stringent scrutiny should be conducted on future iterations of DDD-PIOP that utilize larger patient cohort sizes. To our knowledge, the current 66 patients in this work is one of the largest cohorts in PET-based image outcome studies. The cohort was assigned into training group, validation group and independent test group following the classic group assignment conventions in most deep-learning works. In this work, the training/validation ratio was optimized to ensure the best performance of DDD-PIOP, and the independent test assignment ensures the most robust tests of the DDD-PIOP. Ideally, a larger cohort size will be favored to include more independent tests; however, like most other similar works, limited resources and the retrospective nature make it challenging to find a sufficiently large patient cohort size. Although no statistical tests were reported due to the limited statistical power with five sample size, the presented results could serve as the feasibility support of DDD-PIOP concept and overall workflow design. We expect to investigate DDD-PIOP at other potential anatomical sites with active adaptive PET imaging protocols: based on the current DDD-PIOP framework, PET image predictions by DDD-PIOP would be compared against the actual intra-RT scans to verify the effectiveness of pre-RT plan optimization; at the same time, newer versions of DDD-PIOP with larger training cohort sizes would be in progress.

The most prominent innovation that distinguishes DDD-PIOP from other image-based outcome prediction work is the inclusion of spatial dose distribution as input. The spatial dose distribution includes patient-specific treatment information; to predict patient-specific treatment outcome, the inclusion of patient-specific treatment information is conceptually appealing. Compared to the commonly adopted prescription dose level, the use of spatial dose distribution can potentially capture radiation-induced response with spatial heterogeneity. To further study the role of spatial dose distribution and its impact on DDD-PIOP performance, a comparison study was added. In this study, DDD-PIOP was modified to exclude spatial dose distribution and use PET/CT images only as input, while all other designs, including major CNN architecture and loss function formula, remain unchanged. The comparison results of 2D gamma test passing rates are presented in Table 4. As can be seen, when gamma tests were performed within whole body area, the inclusion of dose information (as in DDD-PIOP) led to improved passing rate results. However, when gamma tests focused on GTV/CTV regions, slightly improved gamma test passing rate results were found when dose information was omitted. Nevertheless, the numerical differences were small, and they did not provide substantial support for either approach. The observed results in Table 4 may be contributed by dose calculation and dose map resampling uncertainties; however, these uncertainties should be small as the same dose calculation setting was used in all 66 cases. A probable theory that may better explain Table 4 results would be the consistency in our current clinical practice. The planned spatial dose distributions that were used for training DDD-PIOP were obtained from clinical plans. These plans followed our institutional planning guidelines that emphasize dose uniformity within the target regions, particularly the primary PTV/CTV regions with large volumes. In other words, all CTVs can be assumed with a uniform 24-Gy dose distribution as the optimal clinical practice. Thus, the pattern variation within the GTV/CTV regions may not be prominent enough to contribute to target region prediction results. On the other hand, dose variations outside GTV/CTV regions were more prominent, and the inclusion of dose distribution brought in additional information for image outcome prediction. Since the body areas contain more voxels than GTV/CTV regions, the slight improvement of gamma test passing rates can be interpreted as the fact that dose inclusion improved prediction accuracy in non GTV/CTV voxels. Generally, the current results did not discourage the conceptually appealing approach of spatial dose distribution inclusion in DDD-PIOP.

**TABLE 4:** 2D gamma test results w/and w/o dose inclusion using different acceptance criteria and evaluation ranges.

	5%/5 mm		10%/5 mm		5%/10 mm		10%/10 mm	
	w/dose (%)	w/o dose (%)	w/dose (%)	w/o dose (%)	w/dose (%)	w/o dose (%)	w/dose (%)	w/o dose (%)
Body	74.5	73.2	80.4	78.7	92.1	92.0	94.3	93.8
GTV	79.2	80.5	83.2	84.8	95.9	97.6	97.0	98.4
CTV	76.4	77.5	82.0	82.4	93.2	97.1	95.2	98.1

To concur with the discussion in the last paragraph, it will be of great interest to extend the DDD-PIOP study to other treatment sites for which PET imaging is involved. In particular, stereotactic body radiotherapy (SBRT) applications of DDD-PIOP will be promising as SBRT usually has heterogeneous dose distributions within the target regions (27). Preclinical studies using small animal experiments will be essential to fully understand DDD-PIOP’s rationale and robustness. One unavoidable limitation of using clinical cases for AI training is the rather limited diversity of training cases: clinical cases should follow current practice guidelines and thus should have overall high quality. On the other hand, an AI should “see all kinds of cases,” including both good cases and suboptimal cases. Otherwise, an AI agent trained by only good cases will be less likely to perform well when encountering an unprecedented “bad” input (28). As a result, small animal experiments which simulate radiotherapy treatments with both “good” and “bad” qualities can increase the robustness of DDD-PIOP towards common clinical application. In addition, like many other deep learning applications in medicine, the implementation of CNN is still a “black box” execution: the function/effect of each convolutional operation has yet to be understood without direct interpretation within the medical study framework. In other words, the actual intelligibility in the developed AI agent remains unclear. Small animal experiments with a larger amount of sample cases provide opportunities to study CNN intelligibility in an analytical manner.

## Conclusion

In this work, an AI agent, DDD-PIOP, was successfully developed for PET image-based outcome prediction of oropharyngeal IMRT using pre-treatment information. DDD-PIOP innovatively includes planned spatial dose distribution and implements a deep learning approach. Preliminary results demonstrated that DDD-PIOP could successfully predict post-radiotherapy PET images with promising quantitative accuracy. DDD-PIOP would be a powerful tool for IMRT dose escalation/reduction during treatment planning to maximize individual IMRT outcome. Future developments of DDD-PIOP will be important before its large-scale clinical application.

## Data Availability Statement

This work is based on the data from a clinical trial. Raw patient data, including all patient medical images, will not be available to the public. Other types of data, including model evaluation results, statistics of image analysis results and model core algorithms, will be available to the public by request to the corresponding author.

## Ethics Statement

The studies involving human participants were reviewed and approved by Duke University Medical Center. The patients/participants provided their written informed consent to participate in this study.

## Author Contributions

All authors contributed in the study design, reviewed the manuscript, and approved submission. CW, DB, and YM contributed in the data collection. CW, JZ, and YS conducted the data processing. YCu conducted the data validation. CW, CL, KL, YCh, and F-FY conducted the data analysis and modeling. CW, CL, JZ, and YS wrote the manuscript.

## Conflict of Interest

The authors declare that the research was conducted in the absence of any commercial or financial relationships that could be construed as a potential conflict of interest.
